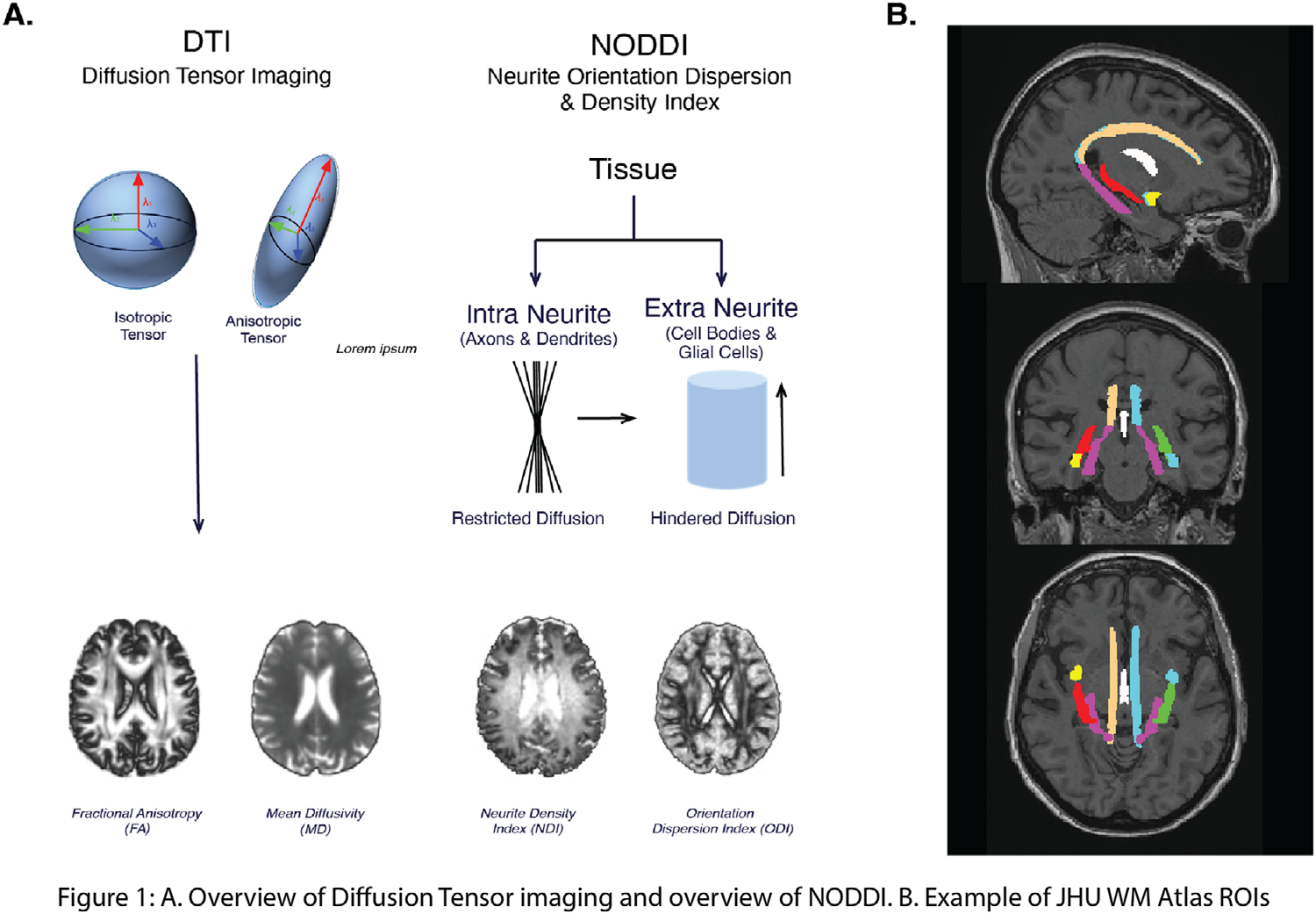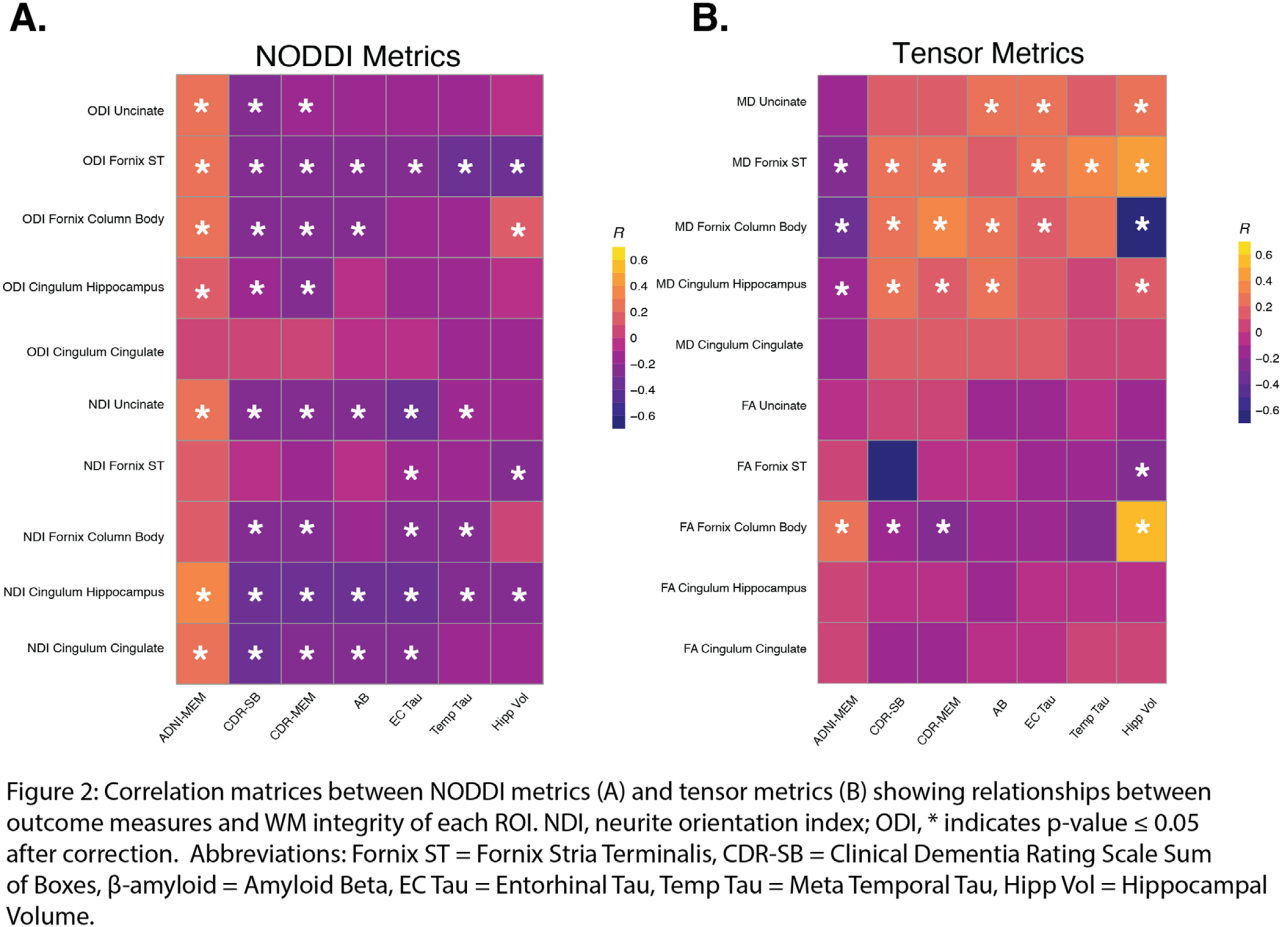# NODDI measures of microstructural integrity in medial temporal lobe white matter pathways are associated with Alzheimer's disease pathology and cognitive outcomes

**DOI:** 10.1002/alz.093986

**Published:** 2025-01-09

**Authors:** Dana M Parker, Jenna N. Adams, Soyun Kim, Liv McMillan, Michael A. Yassa

**Affiliations:** ^1^ University of California, Irvine, Irvine, CA USA

## Abstract

**Background:**

Diffusion tensor imaging has traditionally been used to assess white matter (WM) integrity in Alzheimer’s disease (AD). However, the tensor model is limited in modeling complex WM structure. Neurite Orientation Dispersion and Density Imaging (NODDI), a cutting‐edge technique applied to multishell diffusion MRI, can offer more precise insights into microstructural features of WM integrity. We assessed whether NODDI more sensitively detects AD‐related changes in medial temporal lobe (MTL) WM than traditional tensor metrics.

**Method:**

199 older adults with multishell diffusion MRI from ADNI3 (mean age = 75; 60% female; cognitively unimpaired, n=121; cognitively impaired MCI/dementia, n=78) were analyzed. Tensor metrics of fractional anisotropy (FA) and mean diffusivity (MD), as well as NODDI metrics of Neurite Density Index (NDI) and orientation dispersion Index (ODI), were calculated for MTL WM tracts (JHU Atlas: hippocampal cingulum, fornix column/body, fornix/stria terminalis, and uncinate; Figure 1). A subset of participants received 18F‐florbetapir or 18F‐florbetaben to measure Aß (n = 146; converted to Centiloids), 18F‐flortaucipir to measure tau (n=135), and neuropsychological testing including the Clinical Dementia Rating Sum of Boxes (CDR‐SB) and memory composite score (ADNI‐MEM).

**Result:**

NODDI measures in MTL tracts were more strongly correlated with cognitive performance and AD pathology than standard tensor measures (Figure 2). For example, entorhinal tau was strongly associated with NDI in the cingulum hippocampus (r=‐0.375; p<0.001) and the uncinate (r=‐0.373; p<0.001), and with ODI in the fornix ST (r =‐0.288; p=0.004). Both ODI and NDI across the majority of tracts were associated with CDR‐SB and ADNI‐MEM (see Figure 2). In contrast, FA in any MTL tract was not significantly correlated with either tau or global amyloid‐beta, while MD in MTL tracts showed limited correlations with pathology or cognition.

**Conclusion:**

NODDI metrics offer additional insights about MTL WM integrity in AD that were previously missed due to the limitations of DTI analyses. These results highlight the sensitivity of NODDI applied to multishell diffusion MRI acquisitions to further investigate how WM degeneration contributes to cognitive decline.